# *In silico* profiling of nonsynonymous SNPs of fat mass and obesity-associated gene: possible impacts on the treatment of non-alcoholic fatty liver disease

**DOI:** 10.1186/s12944-023-01782-7

**Published:** 2023-01-30

**Authors:** Damini Patnaik, Atala Bihari Jena, Rout George Kerry, Asim K. Duttaroy

**Affiliations:** 1 Post Graduate Department of Biotechnology, Utkal University, Bhubaneswar, 751004 Odisha India; 2 Department of Neurosurgery, Brigham and Women’s Hospital, Harvard Medical School, Boston, MA 02115 USA; 3 Department of Nutrition, Institute of Basic Medical Sciences, Faculty of Medicine, University of Oslo, Oslo, Norway

**Keywords:** Fat mass and obesity-associated gene (FTO), Nonalcoholic fatty liver disease (NAFLD), Lipogenesis, Genetic polymorphism, FTO ns-SNPs, Nonalcoholic steatohepatitis (NASH)

## Abstract

**Background:**

Nonalcoholic fatty liver, or NAFLD, is the most common chronic liver ailment. It is characterized by excessive fat deposition in hepatocytes of individuals who consume little or no alcohol and are unaffected by specific liver damaging factors. It is also associated with extrahepatic manifestations such as chronic kidney disease, cardiovascular disease, and sleep apnea. The global burden of NAFLD is increasing at an alarming rate. However, no pharmacologically approved drugs against NAFLD are available owing to their complex pathophysiology. Genome-wide association studies have uncovered SNPs in the fat mass and obesity-associated gene (FTO) that are robustly associated with obesity and higher BMI. The prevalence of NAFLD increases in parallel with the increasing prevalence of obesity. Since FTO might play a crucial role in NAFLD development, the current study identified five potentially deleterious mutations from 383 ns-SNPs in the human FTO gene using various in silico tools.

**Methods:**

This study aims to identify potentially deleterious nonsynonymous SNPs (ns-SNPs) employing various in silico tools. Additionally, molecular modeling approaches further studied the structural changes caused by identified SNPs. Moreover, molecular dynamics studies finally investigated the binding potentials of the phytochemicals resveratrol, rosmarinic acid, and capsaicin with different mutant forms of FTO.

**Results:**

The current investigation has five potentially deleterious mutations from 383 ns-SNPs in the human FTO gene using various in silico tools. The present study identified five nsSNPs of the human gene FTO, Gly103Asp, Arg96Pro, Tyr295Cys, and Arg322Gln, with an apparent connection to the disease condition. Modulation of demethylation activity by phytomolecule scanning explains the hepatoprotective action of molecules. The current investigation also suggested that predicted mutations did not affect the binding ability of three polyphenols: rosamarinic acid, resveratrol, and capsaicin.

**Conclusion:**

This study showed that the predicted mutations in FTO did not affect the binding of three polyphenols. Thus, these three molecules can significantly aid drug development against FTO and NAFLD.

**Supplementary Information:**

The online version contains supplementary material available at 10.1186/s12944-023-01782-7.

## Introduction

• Fat Mass and Obesity Associated Gene/FTO, which codes for nucleic acid demethylase, have a robust association with obesity and higher BMI.
• Mutation at the Arg96 position to His results in loss of the demethylase activity of FTO, therefore reducing the catalytic activity of FTO.
• Molecular docking and dynamics have indicated that phytomolecules can bind to the catalytic site of FTO.
• Modulation of demethylation activity by phytomolecule scanning explains their hepatoprotective action.
• Epidemiological evidence suggests a close association of FTO with the development of NAFLD.

Nonalcoholic fatty liver disease (NAFLD) is a metabolic disorder identified by excessive fat deposition in the hepatocytes of individuals who consume little or no alcohol and are unaffected by other specific liver-damaging factors. Although the etiology of NAFLD is not entirely understood, primary risk factors include features of metabolic syndrome such as obesity, type 2 diabetes, and dyslipidemia. NAFLD development is attributed to a sedentary lifestyle, a high-calorie diet, genetic and epigenetic influences, and environmental factors such as the gut microbiome [1]. NAFLD can also lead to a progressive liver disease called nonalcoholic steatohepatitis (NASH), marked by hepatocyte ballooning and inflammation and, in severe conditions, progression to fibrosis, cirrhosis, and hepatocellular carcinoma [2–4]. Genome-wide association studies (GWAS) have uncovered genes robustly associated with NAFLD. These include PNPLA3, transmembrane 6 superfamily member 2 (TM6SF2), glucokinase regulator (GCKR), membrane-bound O- acyltransferase domain-containing 7 (MBOAT7), and hydroxysteroid 17β- dehydrogenase (HSD17B13) [5]. With approximately a quarter of the world’s population affected by NAFLD, it is estimated to become the foremost cause of liver-related morbidity and mortality within the next two decades [6].

The fat mass and obesity-associated (FTO) gene, discovered during a GWAS in 2007, has a robust association with obesity and higher BMI [7]. Epidemiological evidence suggesting a close association of NAFLD with obesity suggests the potential role of FTO in the development of NAFLD [8]. Upregulated liver FTO expression was observed in the NAFLD rat model [9]. In addition, increased serum FTO expression was reported in NAFLD patients, with greater prominence in patients with NASH [10]. FTO is described as the "master switch" because it influences epigenetic control over regulatory pathways in energy homeostasis [11]. FTO shares maximum sequence and structure homology with the *E. coli* DNA repair protein AlkB and its mammalian homologs, including ABH 2 and 3. These proteins are Fe^2+−^ and 2-oxoglutarate (2OG)-dependent dioxygenases [12]. FTO encodes a nucleic acid demethylase in the nucleus, preferentially demethylating 3-meT in ssDNA and m^6^A and 3-meU in ssRNA [13]. RNA modifications affect splicing, intracellular transport, translation, and the half-life of RNA [14]. In addition to energy homeostasis, FTO shows pleiotropic effects on cell proliferation and brain function [15].

Numerous wet lab studies on FTO genes immensely contribute toward the general as well as molecular pathophysiology of the disease. However, despite those studies, a promising therapeutic intervention is far from reach, which begs the requirement of a more sophisticated approach that could use high-throughput data for advanced critical analysis of the disease. This approach is possible with evolving *in silico* approaches that provide statistically significant results with visually vibrant and easy-to-understand data with a very low false discovery rate. Recently, Kumar et al., (2022) [16] displayed via *in silico* analysis the mutational profile of the FTO gene and explained how it affects protein structure and function. In detail, they analyzed the FTO gene's mutational profile and monitored the influence of nonsynonymous (missense) mutations on the structure, dynamics, conformation, and substrate binding of the FTO protein. Through web-based bioinformatic tools, Musliji et al., Field (2021) assessed the involvement of four genes contributing to obesity [17]. The sequences of four so-called obesity genes were gathered and analyzed, including FTO (fat mass and obesity-associated protein), PPARG (peroxisome proliferator-activated receptor), ADRB3 (adrenergic receptor 3), and FABP2 (fatty acid-binding protein 2). Previously, Kumar and Mahalingam, (2018) [18], sought to identify nonsynonymous SNPs (nsSNPs) having the highest projected negative effect on neuronal growth regulator 1 (NEGR1) protein function. They employed five computational tools to forecast the deleterious and pathogenic NEGR1 nsSNPs: PolyPhen, SIFT, PROVEAN, MutPred, and M-CAP.

**Fig. 1:**
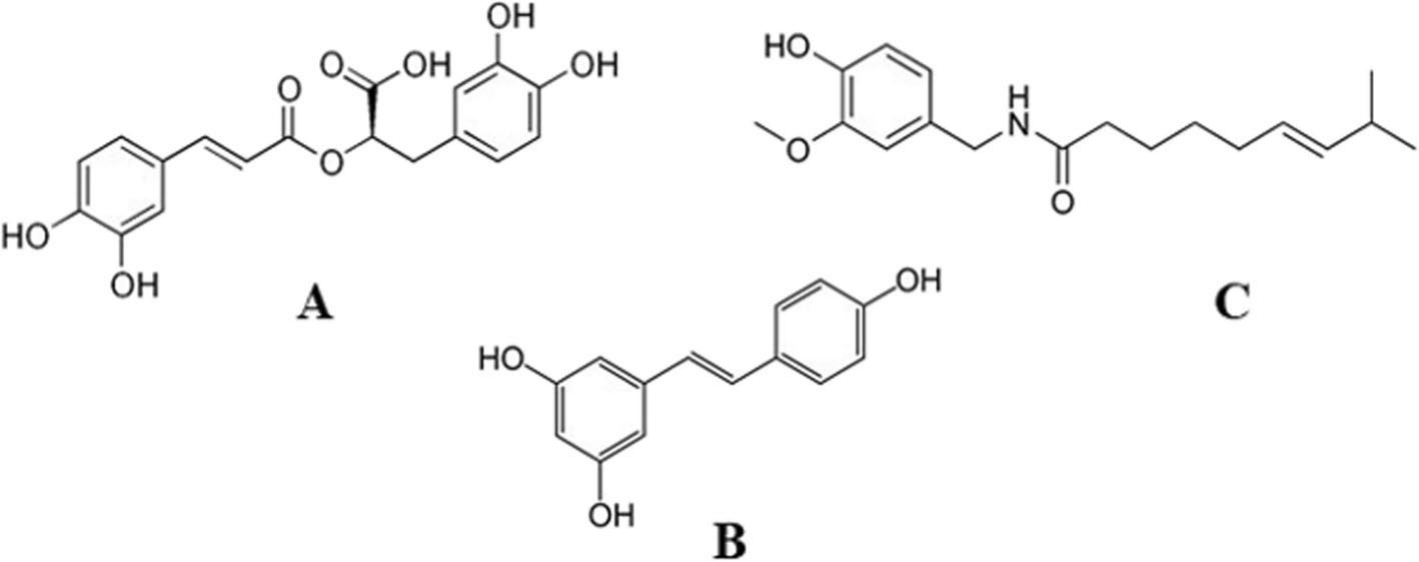
Structure of (**A**) rosamarinic acid, (**B**) resveratrol and (**C**) capsaicin

Despite identifying various risk factors and associated genes, no pharmacologically approved medications are available to treat NAFLD, owing to the complex and multifactorial pathophysiology of the disease [1]. Although promising drugs are in the pipeline for treating NAFLD/NASH, lifestyle intervention remains the front-line treatment [19, 20]. It has been shown that the Mediterranean diet can decrease liver fat content and decrease hepatic steatosis [21, 22]. This is primarily attributed to the antioxidant properties of polyphenolic phytochemicals in the Mediterranean diet [23]. Resveratrol in grapes improves hepatic damage in NAFLD patients [24]. Rosamarinic acid obtained from various plants of the Lamiaceae family has shown potency against NAFLD through an antioxidative mechanism in HepG2 cells [4]. Dietary intake of capsaicin has also been reported to prevent lipid deposition in HepG2 cells and liver tissues through TRPV1 activation-mediated lipolysis (Fig. 1).

In hepatic disorders, several factors are involved, including the creation of reactive oxygen species (ROS), lipid peroxidation, peroxynitrite formation, complement factors, and proinflammatory agents such as chemokines and cytokines [25]. Several biological activities have been reported for rosamarinic acid, capsaicin, and resveratrol. Rosamarinic acid is an ROS scavenger and lipid peroxidation inhibitor [26]. It also possesses anti-inflammatory, angiogenic, and neuroprotective effects. Surprisingly, decreasing the synthesis of IL-6, TNF-alpha, monocyte chemoattractant protein 1, and cyclooxygenase can reduce fat-induced inflammation in adipose tissue. Adiponectin expression is also increased by capsaicin. Resveratrol may also have hepatoprotective effects in NAFLD [27]. It improves lipid and carbohydrate metabolism by reducing hepatic lipid buildup. In addition, resveratrol effectively enhances insulin sensitivity. Several phytochemicals benefit obesity and NAFLD; however, the mechanisms are still unknown. Some compounds bind to FTO protein and thus inhibit the demethylase activity of FTO [28, 29]. Quercetin binds strongly and inhibits FTO activity [30]. These findings open a new avenue toward developing therapeutic interventions through alterations in FTO activity. Based on these findings, it is rational to hypothesize that the selected phytochemicals could be a beneficial therapeutic intervention targeting metabolic abnormalities in NAFLD. The FTO gene is associated with NAFLD patients and animal models [9, 10, 31]. Polymorphism of the FTO gene is also associated with hepatic fat content [11]; therefore, the current study investigated potentially deleterious nonsynonymous SNPs (ns-SNPs) employing various *in silico* tools. The structural changes caused by the identified SNPs were studied using molecular modeling approaches. Furthermore, molecular docking and dynamics studies examined the binding potential of the phytochemicals resveratrol, rosmarinic acid, and capsaicin with different mutant forms of FTO.

Several studies have identified phytochemicals that inhibit the demethylation activity of FTO and their impact on the wild-type FTO structure [32]. However, the effects of the inhibitors on the mutant FTO structure have not been investigated. Therefore, molecular docking was used to study the affinity of the polyphenols with native and wild-type FTO to identify any possible variation in the binding affinity of the selected phytochemicals and mutant protein.

## Materials and methods

### SNP retrieval

**Fig. 2:**
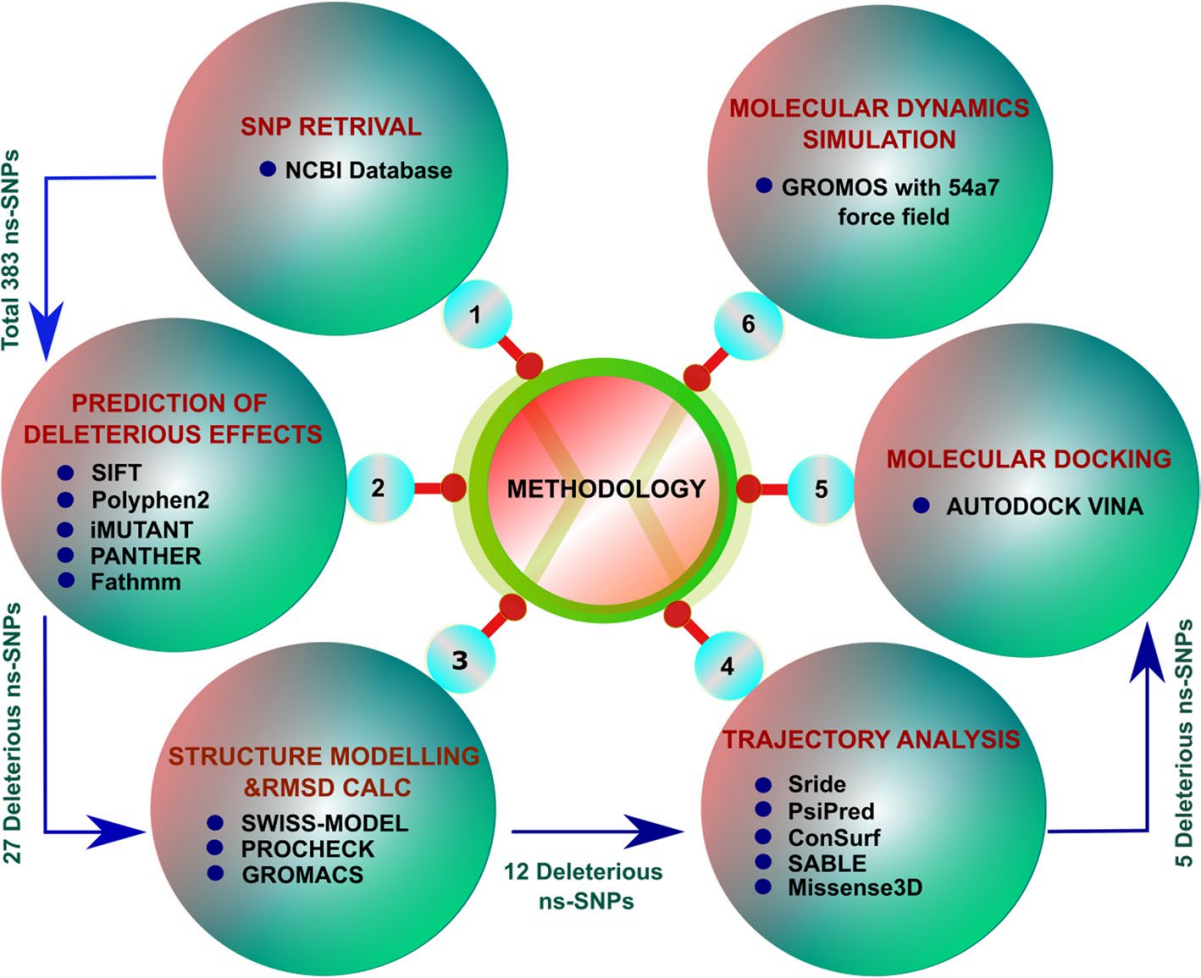
Overview of the methodologies used

The SNPs were retrieved from the SNP database of the National Center for Biotechnology Information (NCBI) (http://www.ncbi.nlm.nih.gov/snp) with various limits (Homosapiens, missense) [33]. An overview of the methodology is shown in Fig. 2.

### Predictions of deleterious effects of FTO

*In silico* tools were employed to distinguish detrimental SNPs from the retrieved SNPs for further analysis. The tool SIFT (Sorting intolerant from tolerant) uses sequence homology to predict whether an amino acid substitution will alter the protein function [20]. The substitution was considered deleterious if the normalized value was less than the cutoff value of 0.05. Polymorphism phenotyping (PolyPhen 2.0) is a tool that recognizes the possible result of amino acid substitutions based on structure and function. The mutations are categorized as ‘probably damaging,’ ‘possibly damaging,’ and ‘benign’ based on the false-positive rate (FPR) threshold [34]. Single-site mutations can lead to changes in protein stability. The protein stability change can be predicted by I-Mutant [35]. The output provides the difference in Gibbs free energy values (DDG/DDG) between mutated and native types (kcal/mol). Protein analysis through evolutionary relationships (Panther) classifies proteins according to family, subfamily, and molecular functions [36]. In addition, functional analysis was performed using Hidden Markov Models v2.3 (FATHMM). FATHMM, a high-throughput web server, was used. This can predict the functional consequences of both coding variants, i.e., nonsynonymous single nucleotide variants (ns-SNVs), and noncoding variants in the human genome, classifying the substitutions as tolerated or damaging [37].

### Structure modeling and root mean square deviation (RMSD) calculations

Mutations can significantly change the structure and stability of proteins. Three-dimensional structure analyses were performed between the native and mutant proteins to evaluate the changes. SWISS-MODEL, an automated homology modeling software, was used to model the 3D structure of the protein. PROCHECK evaluates the stereochemical quality of a protein structure by generating a series of PostScript graphs that analyze its overall and residue-by-residue geometry. Then, the mutants were generated through Swiss PDB Viewer [38]. The energy of both native and mutant proteins was minimized using NOMAD-Ref, a GROMACS-based default tool for minimization that performs calculations of functionally relevant movements in biological macromolecules [39]. Finally, the differences in total energy were computed using the minimized structures. Finally, Swiss PDB Viewer was used to compute the root mean square value deviation (RMSD) between the Natives and mutants.

### Trajectory analysis

The stabilizing residues are defined by SRide based on their long-range interactions, hydrophobicity, and amino acid residue conservation [40]. Further investigation was performed using PSIPRED, a simple and precise secondary structure prediction method. It analyses the output obtained from PSI-BLAST by including two feed-forward neural networks [41]. The functional regions in proteins were identified by using ConSurf [42]. Apart from these, the Missense-3D predicts structural changes introduced by an amino acid substitution and is applicable to analyzing both PDB coordinates and homolog position-Specifictures [43].

### ADMET analysis

Biochemical absorption, distribution, metabolism, excretion, and toxicity (ADMET) are all critical factors of organic or inorganic compounds that could be used for therapeutic purposes. Therefore, a high-quality drug candidate should be effective against the therapeutic target and have adequate ADMET qualities at therapeutic dosages. Therefore, various bioinformatics tools are used to analyze any compound's ADMET properties. Thus, in the current investigation, SWISS ADME software of the Swiss Institute of Bioinformatics and the pkCSM server were used to estimate individual ADME scores of resveratrol, capsaicin, and rosamarinic acid [44, 45]. The toxicity of the phytocompounds was evaluated by using ProTox-II. ProTox-II predicted various toxicity endpoints, such as the compounds' hepatotoxicity, acute toxicity, carcinogenicity, mutagenicity, cytotoxicity, and immunotoxicity. The similarity between the query molecule’s functional group and those reported and found in the software database forms the basis of prediction.

### Molecular docking

FTO’s crystal structure (PDB ID:4QHO) was retrieved from RCSB for all 5 SNPs and was modeled, in addition to energy minimization, using the SWISS-PDB viewer [46]. The drug molecules capsaicin, rosamarinic acid, and resveratrol were acquired from PubChem (Compound CID: 1,548,943, 445,154, and 5,281,792, respectively) in SDF format converted to PDBQT after the addition of hydrogen using Open Babel. The drug molecules were used as ligands, and the energy-minimized native FTO structure and five mutants harboring the solitary SNPs were used as receptors for the docking studies. Protein preparation for docking was performed using AutoDock Tools (version 1.5.6) (ADT). The PDB structure was converted to PDBQT format after removing crystal water molecules and native ligands, assigning Kollman charges, and adding polar hydrogen. The ligand charges were merged, and nonpolar hydrogens were removed in ADT. The search space was defined according to the binding site of FTO, constituted by the residues Arg95, Tyr108, Asn205, His231, Asp233, Tyr295, His307, Thr320, and Arg322 [13]. Docking was performed using AutoDock Vina (version 1.1.2) [47] with exhaustiveness. The interaction of the drug molecules with the active site was analyzed using Schrödinger Maestro. The binding pose with the best binding affinity and maximum interactions with the active site residues was selected for MD simulations.

### Molecular dynamics simulations

The docked complexes of five FTO mutants and the native protein with the three selected drug molecules capsaicin, resveratrol, and rosamarinic acid were subjected to molecular dynamics simulation using the GROMACS (version 2018.7) software suite by previously described methods [48]. The GROMOS 54a7 force field was used to build the protein topology, while ligand topologies were generated using the PRODRG server [49]. A cubic box was generated extending 10 Å from the protein atoms in all directions and solvated using the SPC three-point water model. The system was neutralized by adding an appropriate number of sodium atoms. Using the steepest descent algorithm with a 1000 kJ/mol/nm convergence criterion, the solvated electroneutral system was energy minimized to eliminate steric clashes. A cutoff of 1.2 nm was used for computing both van der Waals and Coulombic interactions, while long-range electrostatic interactions were handled using particle‒mesh Ewald (PME). The complex's equilibrium was performed first in a canonical ensemble (NVT) by restraining solvent and ions at 300 K for 100 ps. This was followed by NPT equilibration for 100 ps, where the restraint weight on the protein‒ligand complex was gradually reduced. The LINC algorithm was used as the bond constraint algorithm, while constant temperature and pressure were simulated using Berendsen’s thermostat and Parrinello-Rahman pressure coupling [50, 51]. A production simulation was performed for 100 ns with a step size of 2 fs using a leapfrog dynamics integrator. Postsimulation analysis was performed using GROMACS modules and in-house python scripts.

## Results

### Data mining

A total of 383 SNPs were obtained from the NCBI database, with functions limited to *Homo sapiens* retrieved.

### Prediction of deleterious ns-SNPs

Twenty-seven deleterious ns-SNPs were identified by PolyPhen 2.0, SIFT, Panther, FATHMM, and I-Mutant 3.0. Previous studies have indicated that predicting deleterious ns-SNPs by combined tool analysis leads to more accurate results. It was ascertained that only 27 ns-SNPs are expected to be ‘‘possibly deleterious’’ by all tools named in Table S1. Variation in alleles showed 10 ns-SNPs for Adenine → Guanine; 6 for Cytosine → Thymine; 1 for Adenine → Cytosine; 3 for Guanine → Thymine; 5 for Cytosine → Guanine; and 2 for Adenine → Thymine. The Adenine → Guanine nucleotide variation was seen maximum times with a SIFT score of zero, followed by Cytosine → Thymine. This verified the results obtained with the FTO gene analyzed in this study and confirmed that point mutations might be deleterious. The results are shown in Supplementary Table S1.

### Modeling protein structures and RMSD calculation

Point mutations significantly alter the stability of the protein structures. Therefore, it is essential to study the relationship between the structure and function of proteins using three-dimensional structures [52]. The total energy of the native type was -30, 249 kJ/mol. Considerable variations were found in the total energy of 25 mutants compared to the native type. The structure of the modeled mutant proteins is shown in Supplementary Figure S1.

An *in* *silico* study on the FTO gene predicted that ns-SNPs, with RMSD > 0.3, play a substantial role in the commencement of disease. Swiss PDB Viewer was used to computing RMSD values. The present study found 12 mutant models with deviations greater than 0.3A. A higher RMSD value indicates more deviation between the native and mutant structures. This variation is damaging, as it leads to divergence in the 3-D space of the binding site of FTO genes. This, in turn, leads to a change in the critical efficiencies of the inhibitors. Hence, these 12 ns-SNPs (Table S2) can be expected to be ‘‘deleterious’’ and were selected for further analysis.

### Trajectory analysis

Stabilizing residues for deleterious ns-SNPs were identified and compared with native structures. Panther was used to identifying destabilizing residues in the ns-SNPs. Destabilizing residues were present in five out of the twelve ns-SNPs. The added residues with negative free energy (DDG) values were categorized as “destabilized.” For the five ‘‘highly deleterious ns-SNPs,’’ with RMSD > 0.3 and destabilizing residues in proteins, solvent accessibility and secondary structure predictions were carried out through SRide, PSIPRED, ConSurf, and Missense 3D (Table 1).

**Table 1:** Trajectory analysis and stabilizing residues of the deleterious SNPs. Destabilizing residues are shown in bold. (E- Extracellular, C-coil, S-strands)

S.NO	Residual change	SRide(stabilizing residue)	PSIPRED		ConSurf		Missense3D (structural damage)
			Native (N)	Mutant (M)	Native (N)	Mutant (M)	
1	Native	ILE99, VAL116, TRP118,ASN205, ALA243, ALA311					
2	R96P	ILE99, VAL116, TRP118, ASN205, ALA243, TRP270, ALA311	E	E	9	8	Damaged
3	G103D	ILE99, VAL116, TRP118, ASN205, ALA243, TRP270, ALA311	S	C	9	7	Damaged
4	Y295C	ILE99, VAL116, TRP118, ASN205, TRP270, ALA311	S	S	9	9	Damaged
5	R316Q	ILE99, VAL116, TRP118, ALA243, TRP270, ALA311	E	E	9	9	Damaged
6	R322Q	ILE99, VAL116, TRP118, ASN205, TRP270, ALA311	C	S	9	9	Damaged

### ADMET Analysis

The results of the ADMET analysis are tabulated in Table 2 and Table 3. All three molecules are not substrates for P-gp. The GI absorption is high for capsaicin and resveratrol, whereas it is low for rosamarinic acid (Table 3). Capsaicin and resveratrol are capable of crossing the brain blood barrier (BBB). CYP isoforms (CYP1A2, CYP2C19, CYP2D6, and CYP3A4) and P-gp can synergistically process small molecules [42]. This, in turn, improves tissue and organism protection. Inhibition of these isoenzymes may lead to harmful effects such as drug accumulation and lower clearance of its metabolites. Capsaicin and resveratrol inhibit CYP1A2. Resveratrol also inhibits CYP2C9, whereas capsaicin inhibits CYP2D6. None of the CYP isoforms were inhibited by rosmarinic acid. Capsaicin and resveratrol are CYP3A4 inhibitors. The K_*P*_ or skin permeability coefficient correlates with molecular size and lipophilicity (Table 3) [43]. The skin permeant of a molecule will be greater if the log K_*P*_ value is less negative. Resveratrol with a log K_*P*_ value of -5.47 (cm/s) will have high skin permeability, whereas rosamarinic acid will have the least (-6.82 cm/s). The ProTox-II results showed that resveratrol did not have any possible toxic effects.

**Table 2:** ADME profile of the three phytomolecules

S.No	Toxicity Type	Result		
		**Capsaicin**	**Rosamarinic acid**	**Resveratrol**
1	Hepatotoxicity	Inactive	Inactive	Inactive
2	Carcinogenicity	Active	Inactive	Inactive
3	Immunotoxicity	Active	Active	Inactive
4	Mutagenicity	Active	Inactive	Inactive
5	Cytotoxicity	Inactive	Inactive	Inactive

**Table 3:** Pharmacokinetic properties of the selected molecules capsaicin, rosamarinic acid and resveratrol

**Molecules**	GI absorption	BBB permeant	Pgp substrate	CYP1A2 inhibitor	CYP2C19 inhibitor	CYP2C9 inhibitor	CYP2D6 inhibitor	CYP3A4 inhibitor	log Kp (cm/s)
Capsaicin	High	Yes	No	Yes	No	No	Yes	Yes	-5.62
Rosamarinic acid	Low	No	No	No	No	No	No	No	-6.82
Resveratrol	High	Yes	No	Yes	No	Yes	No	Yes	-5.47

### Molecular docking

**Fig. 3:**
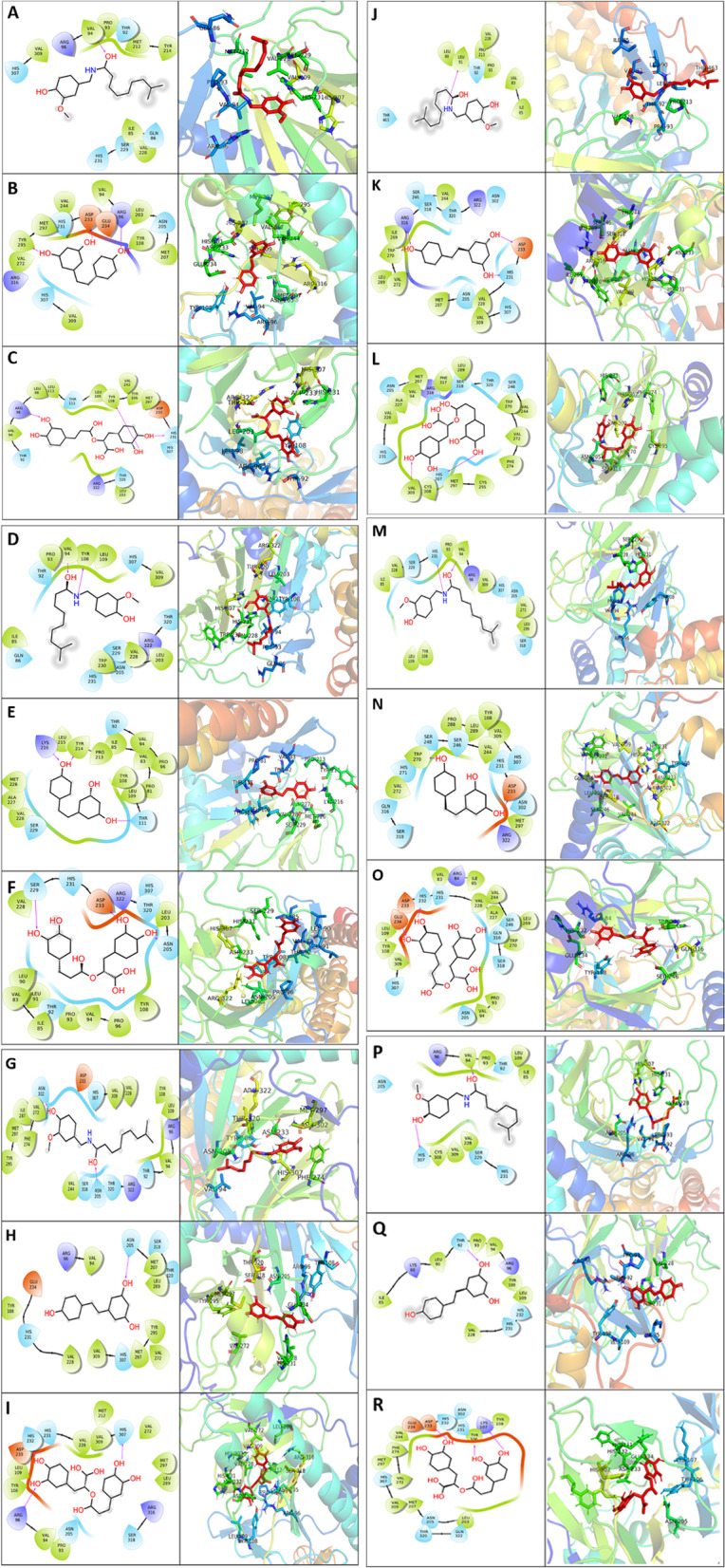
Drug-ligand interaction predicted for FTO and its mutants: (**A**) native FTO with capsaicin, (**B**) native FTO with resveratrol, (**C**) native FTO with rosmarinic acid, (**D**) R96P with capsaicin, (**E**) R96P with resveratrol, (**F**) R96P with rosmarinic acid, (**G**) G103D with capsaicin, (**H**) G103D with resveratrol, (**I**) G103D with rosmarinic acid, (**J**) Y295C with capsaicin, (**K**) Y295C with resveratrol, (**L**) Y295C with rosmarinic acid, (**M**) R316Q with capsaicin, (**N**) R316Q with resveratrol, (**O**) R316Q with rosmarinic acid, (**P**) R322Q with rosmarinic acid, (**Q**) R322Q with resveratrol, and (**R**) R322Q with rosmarinic acid

Molecular docking of the three selected phytochemicals with FTO was performed using AutoDock Vina to predict their binding poses. Rosamarinic acid showed the best binding affinity among all three drugs against native and predicted deleterious mutants of FTO. MD simulations selected the binding pose with the best affinity for further refinement. The predicted binding relationship of drug molecules with FTO native and mutant forms obtained from AutoDock Vina is represented in Table 4. Detailed interactions of the phytomolecules with different mutant forms of FTO are shown in Fig. 3.

**Table 4:** Predicted binding affinity of drug molecules with FTO native and mutant forms obtained from AutoDock Vina

S.No	Target Protein	Predicted Binding Affinity (kcal/mol)		
		**Capsaicin**	**Resveratrol**	**Rosamarinic acid**
**1**	FTO Native	-7.8	-7.8	-9.2
**2**	FTO R96P	-7.7	-7.6	-8.7
**3**	FTO G103D	-7.9	-7.8	-8.9
**4**	FTO Y295C	-8.3	-7.8	-9.3
**5**	FTO R316Q	-8.6	-8.4	-9.2
**6**	FTO R322Q	-7.8	-7.6	-9.7

### Molecular dynamics simulation studies

Molecular dynamics simulations were performed to refine the binding pose and assess the stability of the protein‒ligand complex under aqueous conditions. In addition, the structural perturbations of drug binding to native and mutant forms of FTO were examined using various parameters obtained from the analysis of the 100 ns trajectory.

**Fig. 4:**
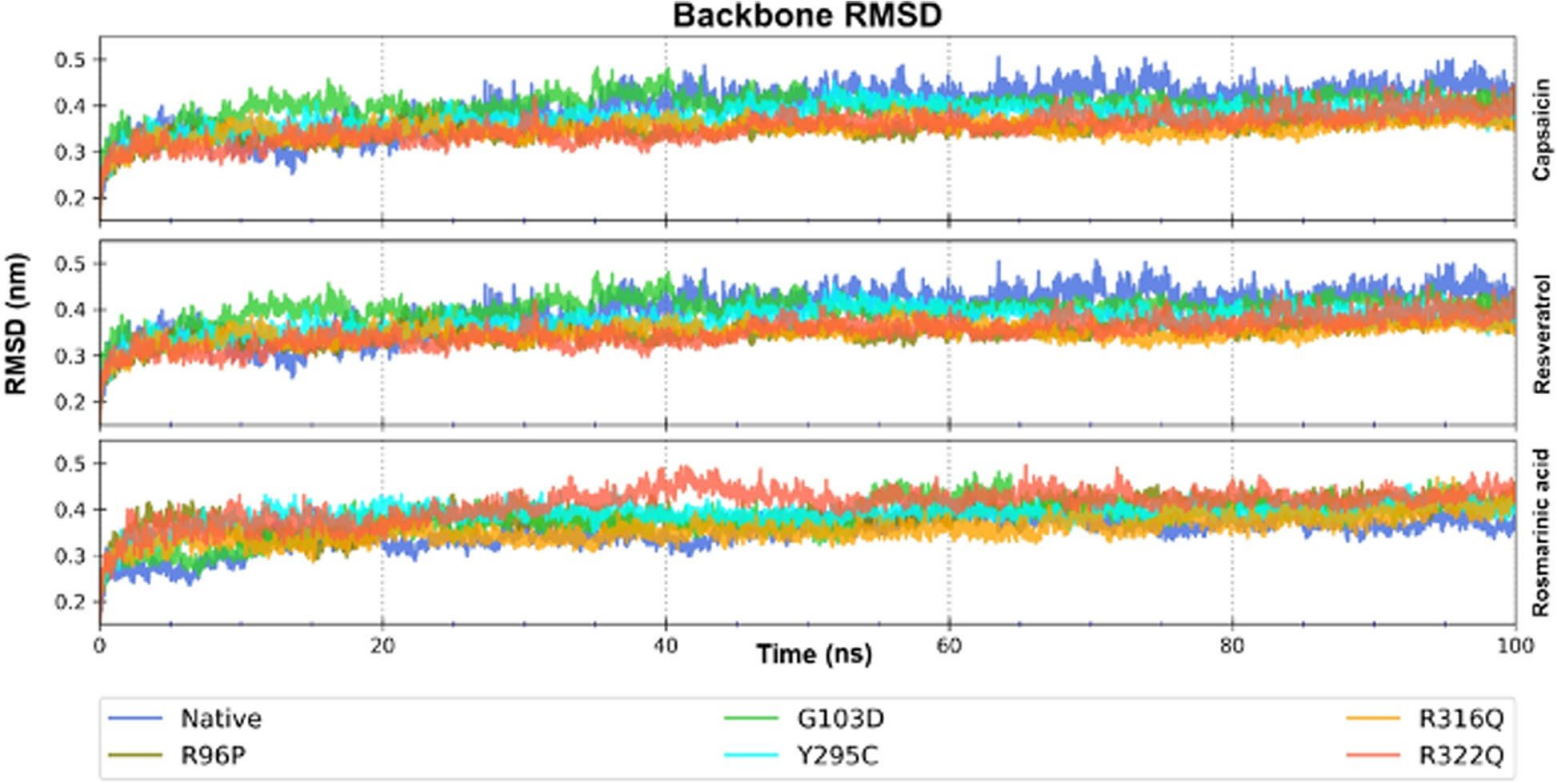
Structure modeling and root mean square deviation (RMSD) profile of backbone coordinates of FTO native and mutant forms with the three molecules for the 100 ns simulation

The root mean square deviation (RMSD) was calculated to quantify the conformational changes in the protein structure. After stabilization, the average protein backbone RMSD was less than 3 nm. The well-equilibrated backbone RMSD profile (Fig. 4) indicates no significant structural perturbations in the presence of all five mutations and drug binding. The structural integrity of FTO mutants and native protein was also evidenced by the radius of the gyration plot (Fig. S2), which indicates the compactness of the protein. The compactness of the protein was marginally higher in the case of rosmarinic acid binding, displaying lower Rg values, followed by resveratrol and then capsaicin.

**Fig. 5:**
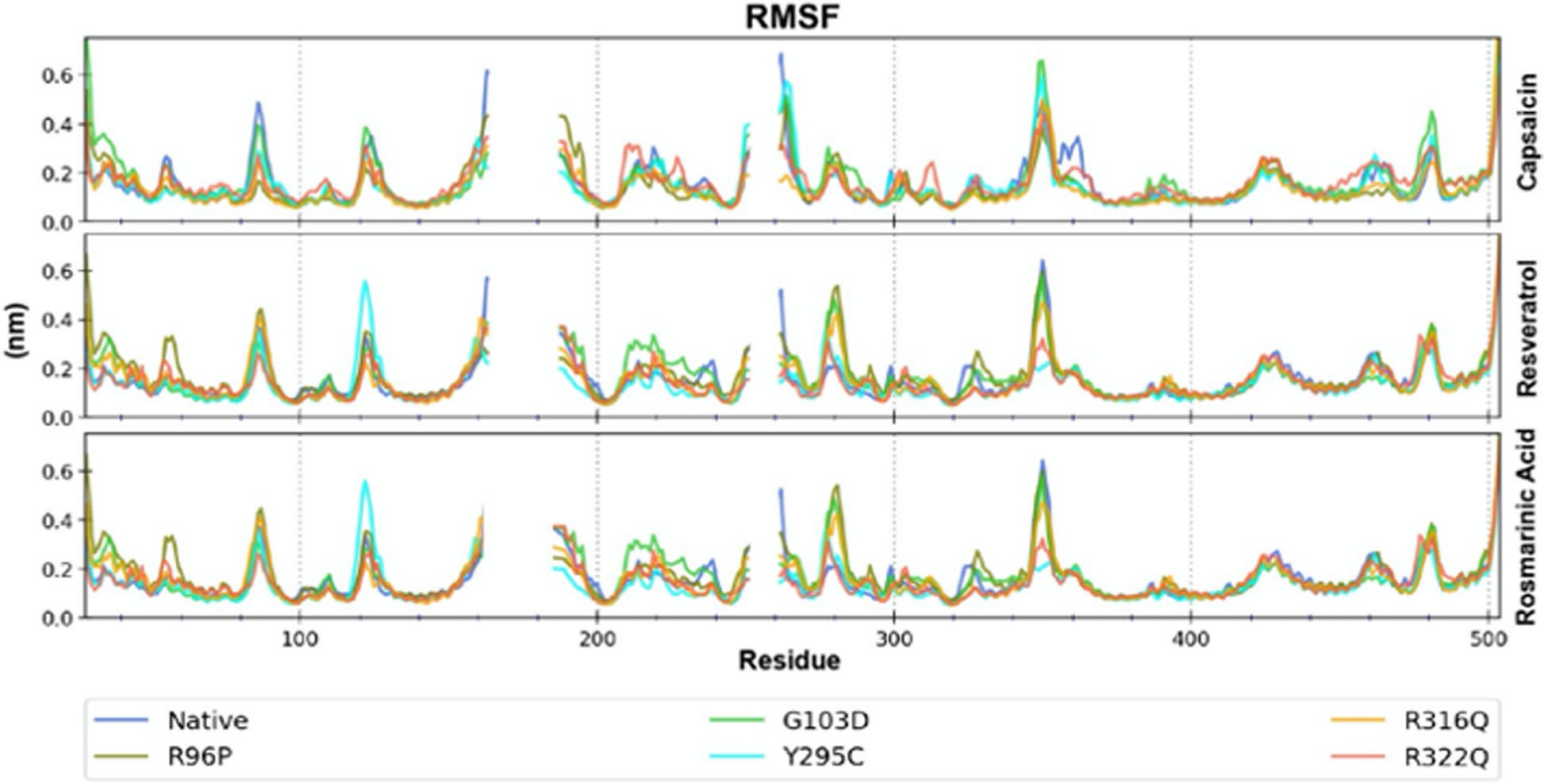
RMSF profile of the mutant and native forms of FTO bound to these molecules

Similarly, more minor changes in SASA with time reflect the global structural stability of the protein since conformational changes are often accompanied by changes in solvent accessibility of the protein. The differences in the SASA profile for different protein complexes resulted from differences in solvent substitution upon drug binding (Fig. S3). The plot of RMSF of Cα atoms throughout the simulations is shown in Fig. 5.

**Fig. 6:**
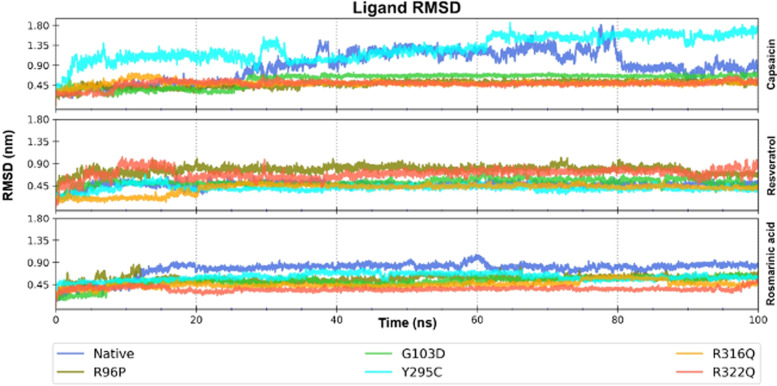
Ligand RMSD profile for the 100 ns simulation

The binding potential of FTO with the three selected molecules shown in docking studies was substantiated in MD simulation studies. The relative stability of the protein‒ligand complexes was assessed from the ligand RMSD profiles (Fig. 6). The complexes of drugs rosamarinic acid and resveratrol with different forms of FTO were stable with well-equilibrated ligand RMSD values throughout the 100 ns. In the case of capsaicin, the complex with Arg96Pro, Gly103Asp, Arg316Gln, and Arg322Gln mutants of FTO showed stable binding through interactions with the residues in the jelly roll motif, while native and Tyr295Cys mutant FTO had comparatively fewer stable complexes with capsaicin.

## Discussion

Despite the elusive molecular mechanism underlying the biology of the FTO gene, it remains a crucial modulator of various diseases, specifically NAFLD. FTO genetic variants with potential disease association in the present study using an *in silico* approach (Table 5). Of the 383 ns-SNPs obtained from dbSNP for the human FTO gene, 27 were predicted to be deleterious using SIFT, PolyPhen 2.0, I-Mutant, Panther, and FATHMM. Furthermore, 12 out of 27 were shortlisted after RMSD calculations. Five final ns-SNPs of the human FTO gene were identified after trajectory analysis using SRide vis-à-vis Arg96Pro, Gly103Asp, Arg316Gln, Tyr295Cys, and Arg322Gln. The clinical significance of some of these mutations is reported based on ACMG classification obtained from Var Some and ClinVar, Table 5 [53, 54].

**Table 5:** Genetic variants of FTO with potential disease association

dbSNP ID	Induced Mutation	Clinical Significance
rs121918214	Arg316Gln	Pathogenic
rs139577103	Arg96Pro	Uncertain Significance
rs745616565	Arg322Gln	Likely Pathogenic
rs1203776934	Gly103Asp	Uncertain Significance
rs1259762053	Tyr295Cys	-

The FTO structure comprises two domains: an N-terminal domain with β-helices in a jelly roll motif that harbors the catalytic core and an α-helical C-terminal domain with unknown structural homology [13]. The residues at positions 96, 103, 295, 316, and 322 are well conserved among all FTO homologs [55]. Tyr295, Arg316, and Arg322 are known to form crucial interactions with the carboxylate of the cosubstrate 2OG, including salt bridges and hydrogen bonding. Arg96 is located on the substrate recognition lid and is responsible for specific substrate base recognition through hydrogen bond interactions. The residue Gly103 is present at the interface of the N-terminal and C-terminal domains. Despite having the catalytic core, the N-terminal domain (NTD) is nonfunctional when expressed in the absence of the C-terminal domain (CTD), or the interface between NTD and CTD is perturbed [56].

The mutation Arg316Gln is known to cause the inactivation of FTO enzymatic activity and cause the autosomal-recessive lethal disorder. Studies using fibroblasts harboring the FTO protein with the Arg316Gln mutation showed enlarged cell size, altered cell morphology, increased senescence, and decreased cell viability [57]. Since the Arg316Gln SNP has been classified as pathogenic, it endorses the pipeline for identifying potentially deleterious ns-SNPs. Similarly, the mutation Arg322Gln also renders FTO inactive and is known to be associated with malformation syndrome, an autosomal recessive disorder. Mutation at the Arg96 position to His results in loss of demethylase activity and a similar effect is thus expected for the conversion to proline [56]. Gly103Asp mutations have shown reduced demethylase activity in AlkB and ABH homologs of FTO. Since aspartic acid is a charged and large amino acid compared to glycine, the structural changes accompanying the mutation can drastically alter the interactions at the NTD and CTD of the FTO interface. The effect of the Tyr295Cys mutation in FTO has not been studied in detail. The change from tyrosine to cysteine at position 295 hampers the hydrogen bond between Tyr295 and 2-OG, reducing FTO activity.

Molecular docking and dynamics studies have shown that rosamarinic acid, resveratrol, and capsaicin show promising effects against NAFLD, as they can potentially bind to the catalytic site of FTO. In detail, the predicted binding affinity was calculated in kcal/mol by docking. Rosamarinic acid is bound to the target native FTO protein with the highest binding affinity of approximately -9.2 kcal/mol. The higher binding affinity was maintained in all the mutated states of the proteins, such as for R96P, G103D, Y295C, R316Q, and R322Q, and the respective binding affinities were -8.7, -8.9, -9.3, -9.2 and -9.7 kcal/mol. Similar results were also observed for resveratrol and capsaicin, as presented in Table 4, suggesting that in addition to native FTO, the mutated forms of the proteins could also be inhibited effectively through natural phytocompounds. Molecular dynamics, on the other hand, resulted in quantifying the conformational changes in the protein structure after the interaction of the compounds rosamarinic acid, resveratrol, and capsaicin with the native form and the mutated versions of the FTO proteins. This suggested that the compounds altered the structural confirmation of both native FTO protein and its mutated forms visualized in the figures. Furthermore, other polyphenolic compounds, such as flavonoids, could interact with FTO. Modulating the demethylation activity by these phytochemicals can explain the hepatoprotective action of these molecules, as the molecules' binding was not affected by the mutations in the FTO gene caused by the predicted deleterious ns-SNPs.

### Study strengths and limitations

The global burden of NAFLD is increasing at an alarming rate. However, no pharmacologically approved drugs against NAFLD are available owing to their complex pathophysiology. Since FTO might play a crucial role in NAFLD development, the current study identified five potentially deleterious mutations from 383 ns-SNPs in the human FTO gene using various in silico tools. The structural effect of the mutations was studied using molecular modeling. The mutations Arg316 and Arg322 have previously been validated through in vitro assays; further validation is required for Arg96Pro, Gly103Asp, and Tyr295Cys.

The current study explored diverse computational techniques (molecular docking and simulation) to establish natural compound-based therapeutic strategies for NAFLD. If the work had been carried out in wet lab experimentation, this would require more sophisticated instrumentation, time, and manpower. Despite the *in silico* efficacy of these phytocompounds, in vitro and in vivo analyses are still required for definitive conclusions. The binding of the three molecules was not affected by the predicted mutations in FTO. Thus, these molecules can significantly aid drug development against FTO and NAFLD. The mentioned compounds have been studied *in silico*, providing evidence of their beneficial effects on liver diseases, such as NAFLD. Although lifestyle modifications involving diet and exercise currently remain the first line of treatment for NAFLD, the compounds reviewed here could also improve NAFLD treatment.

## Conclusion

The global burden of NAFLD is increasing at an alarming rate. However, no pharmacologically approved drugs against NAFLD are available due to its complex pathophysiology. Since FTO might play a crucial role in NAFLD development, the present study identified five potentially deleterious mutations from 383 ns-SNPs in the human FTO gene using various *in silico* tools. The structural effect of the mutations was studied using molecular modeling. Arg316 and Arg322 were previously validated through in vitro assays; further validation is required for Arg96Pro, Gly103Asp, and Tyr295Cys.

Additionally, molecular docking and simulations were used to analyze the potential binding ability of three natural polyphenols, namely, rosamarinic acid, resveratrol, and capsaicin. The binding of the three molecules was not affected by the predicted mutations. Thus, these molecules can significantly aid in drug development against FTO and NAFLD. However, wet lab research should be used to support the study further for higher clinical relevance.

## Supplementary Information

**Additional file 1:** **Table S1.** List of ‘‘possible deleterious ns-SNPs’’by combined prediction. 12 ns-SNPs predicted to be deleterious are in bold. **Table S2.** RMSD Values of Deleterious SNPs. **Figure S1.** 3-Dimensional structure of deleterious ns-SNPs (A) R96P (B) G103D (C) Y295C (D) R322Q (E) R316Q. **Figure S2.** Radius of Gyration plots for mutant andnative forms of FTO bound to the drug molecules. **Figure S3.** Solvent Accessible Surface Area of theprotein-ligand complex throughout the simulation length.

## Data Availability

The datasets and analysis of the current study can be availed upon request.
