# Supplementary material for: In silico profiling of nonsynonymous SNPs of fat mass and obesity-associated gene: possible impacts on the treatment of non-alcoholic fatty liver disease
Source: Lipids Health Dis. 2023 Jan 30;22:17. doi: 10.1186/s12944-023-01782-7 (PMC9885621; doi:10.1186/s12944-023-01782-7)
Supplement: Supplementary file 1 — Additional file 1: Table S1. List of ‘‘possible deleterious ns-SNPs’’by combined prediction. 12 ns-SNPs predicted to be deleterious are in bold. Table S2. RMSD Values of Deleterious SNPs. Figure S1. 3-Dimensional structure of deleterious ns-SNPs (A) R96P (B) G103D (C) Y295C (D) R322Q (E) R316Q. Figure S2. Radius of Gyration plots for mutant andnative forms of FTO bound to the drug molecules. Figure S3. Solvent Accessible Surface Area of theprotein-ligand complex throughout the simulation length. [file 12944_2023_1782_MOESM1_ESM.docx]

SupplementaryFile

*In Silico* Profiling of Non-Synonymous SNPs of *FTO* Gene for Early Diagnosis of Non-Alcoholic Fatty Liver Disease

DaminiPatnaik^1^, Atala Bihari Jena^2*^

^1^PostGraduateDepartmentofBiotechnology,UtkalUniversity,Bhubaneswar-751004,Odisha,India

^2^CentreofExcellenceinIntegratedOmicsandComputationalBiology,UtkalUniversity,Bhubaneswar-751004,Odisha,India

^*^Corresponding Author: Atala Bihari Jena ([jena.atala@utkaluniversity.ac.in](mailto:jena.atala@utkaluniversity.ac.in); [Jena.atala@gmail.com](mailto:Jena.atala@gmail.com))


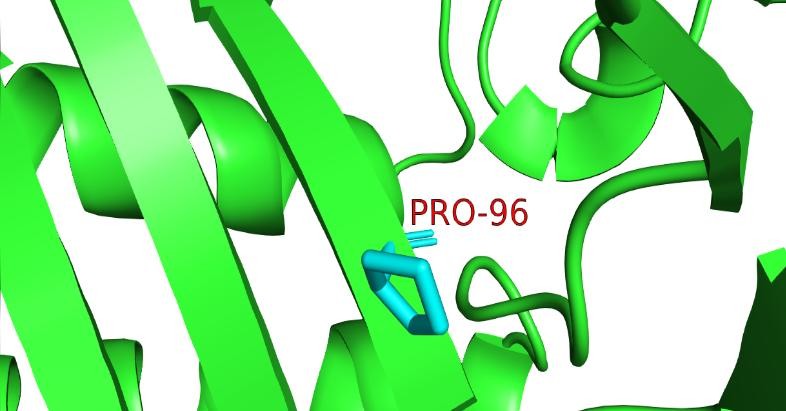

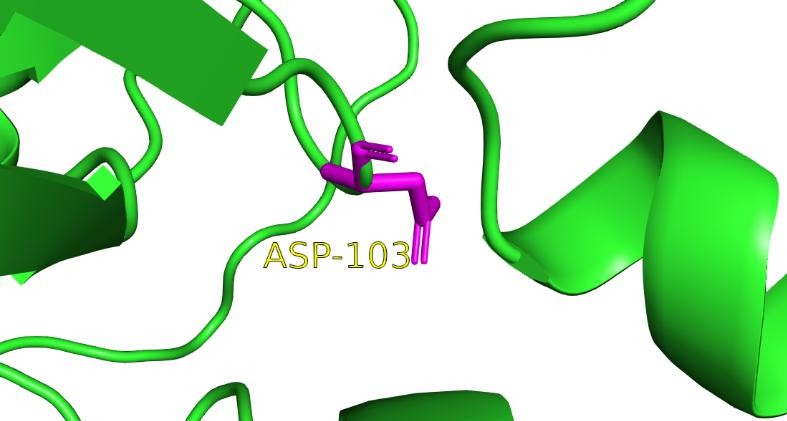

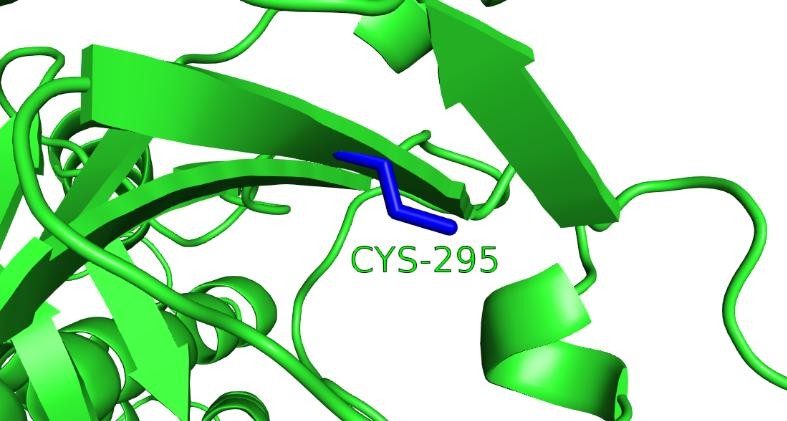

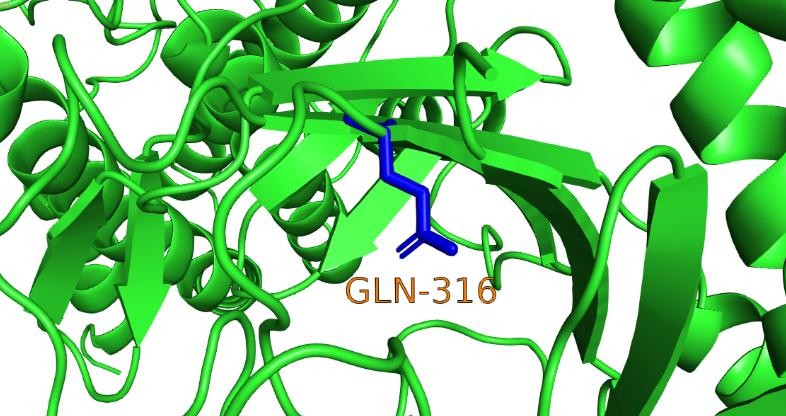

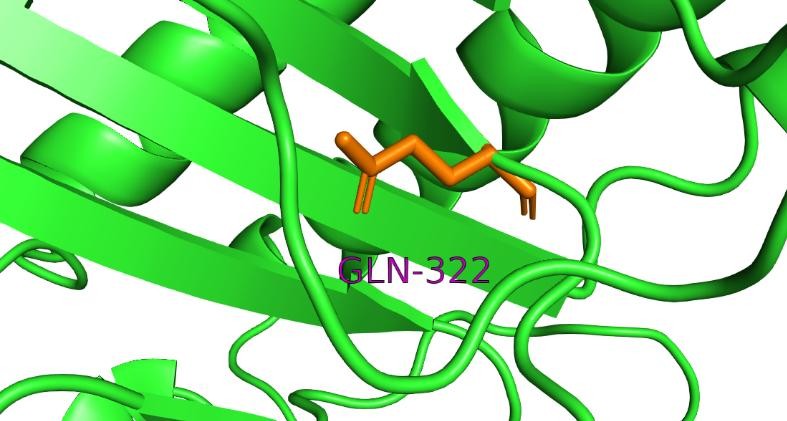


**C**

**E**

**B**

**D**

**A**

**Figure S1:** 3-Dimensional structure of deleterious ns-SNPs (A) R96P (B) G103D (C) Y295C (D) R322Q (E) R316Q.


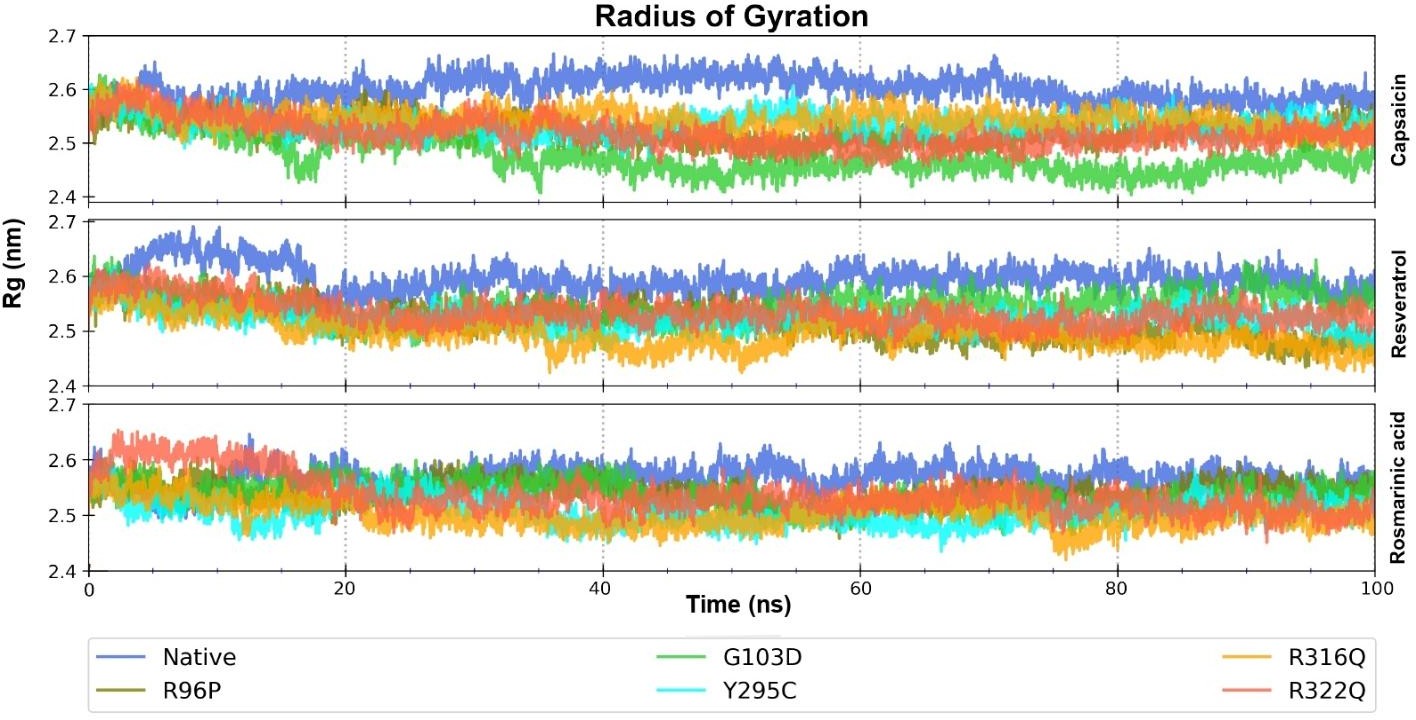


**Figure S2:** Radius of Gyration plots for mutant and native forms of FTO bound to the drug molecules.


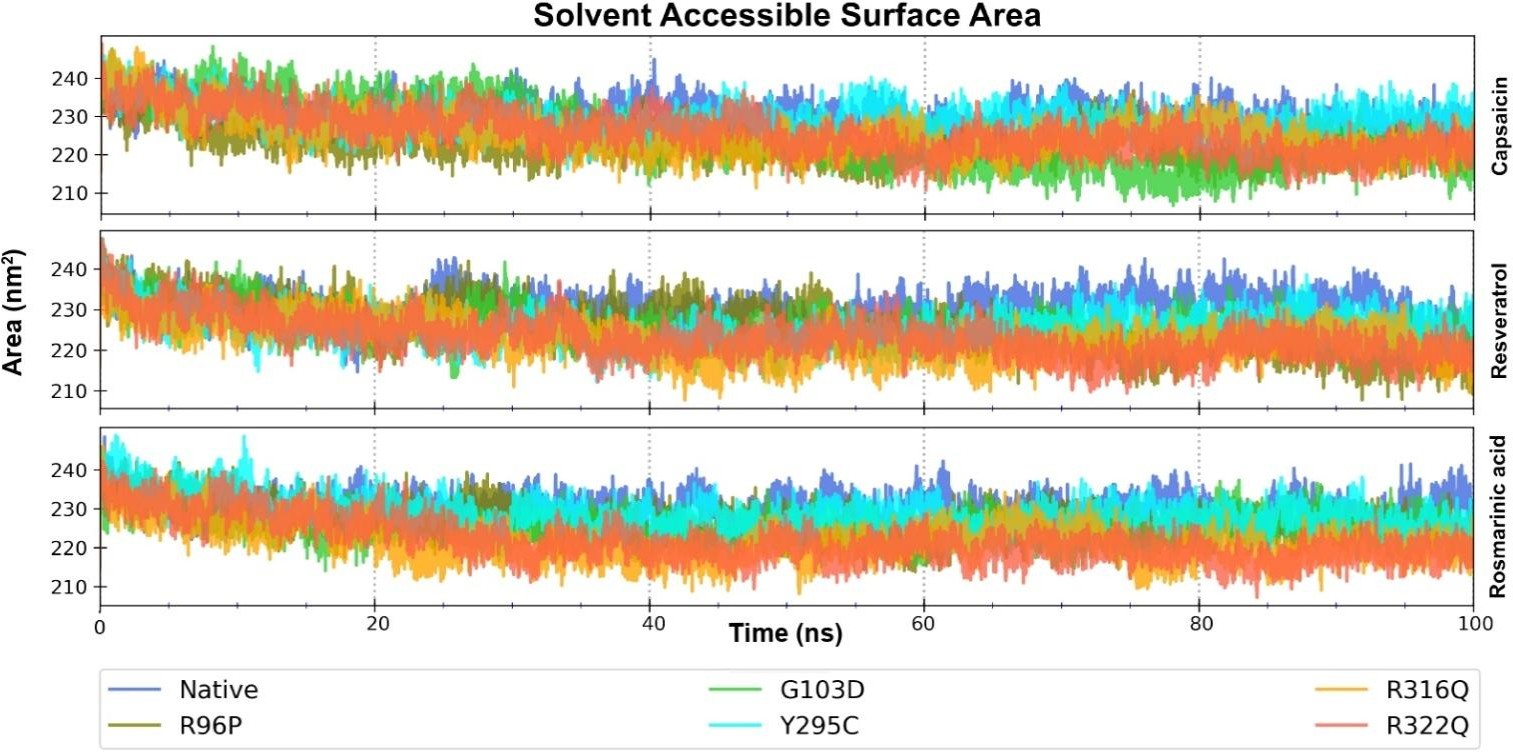


**Figure S3:** Solvent Accessible Surface Area of the protein-ligand complex throughout the simulation length.

**Table S1:** List of ‘‘possible deleterious ns-SNPs’’ by combined prediction. 12 ns-SNPs predicted to be deleterious are in bold.

| **S.No.** | **RsID** | **ALLELE** | **RESIDUAL**  **CHANGE** | **SIFT** | **PolyPhen-2** | **PolyPhen-2** | **I-Mutant30** | **PANTHER** | **fathmm** |
| --- | --- | --- | --- | --- | --- | --- | --- | --- | --- |
|  |  |  |  |  | **HUMANDIV** | **HUMAN**  **VARIANCE** |  |  |  |
| 1 | [rs121918214](https://www.ncbi.nlm.nih.gov/snp/rs121918214) | G/A | **R316Q** | 0 | 0.999 | 0.922 | -0.4 | 1628 | -2.02 |
| 2 | [rs886052104](https://www.ncbi.nlm.nih.gov/snp/rs886052104) | C/T | T320I | 0 | 0.999 | 0.954 | -0.6 | 1628 | -1.82 |
| 3 | [rs138348216](https://www.ncbi.nlm.nih.gov/snp/rs138348216) | A/G | **M207V** | 0 | 0.985 | 0.977 | -1.02 | 1628 | -1.59 |
| 4 | [rs139577103](https://www.ncbi.nlm.nih.gov/snp/rs139577103) | G/C | **R96P** | 0 | 1 | 0.999 | -1.51 | 1628 | -2.06 |
| 5 | [rs139577103](https://www.ncbi.nlm.nih.gov/snp/rs139577103) | G/A | R96H | 0 | 1 | 0.999 | -2.15 | 1628 | -2.05 |
| 6 | [rs147561986](https://www.ncbi.nlm.nih.gov/snp/rs200201735) | A/G | N143S | 0 | 0.999 | 0.997 | -0.54 | 455 | -1.83 |
| 7 | [rs200201735](https://www.ncbi.nlm.nih.gov/snp/rs200201735) | C/G | R322G | 0 | 0.996 | 0.945 | -2.42 | 1628 | -1.7 |
| 8 | [rs370009039](https://www.ncbi.nlm.nih.gov/snp/rs370009039) | A/T | K216N | 0 | 1 | 0.998 | -0.38 | 1628 | -1.62 |
| 9 | [rs373076420](https://www.ncbi.nlm.nih.gov/snp/rs373076420) | C/T | **P288L** | 0 | 1 | 0.999 | -0.32 | 1628 | -2.2 |
| 10 | [rs550932456](https://www.ncbi.nlm.nih.gov/snp/rs550932456) | A/G | H62R | 0 | 1 | 0.998 | -0.32 | 1628 | -1.95 |
| 11 | [rs745616565](https://www.ncbi.nlm.nih.gov/snp/rs745616565) | G/A | **R322Q** | 0 | 0.999 | 0.922 | -1.58 | 1628 | -2.02 |
| 12 | [rs756850408](https://www.ncbi.nlm.nih.gov/snp/rs756850408) | A/G | N200D | 0 | 0.994 | 0.999 | -0.4 | 1628 | -1.82 |
| 13 | [rs757311078](https://www.ncbi.nlm.nih.gov/snp/rs757311078) | C/T | R96C | 0 | 0.996 | 0.999 | -2.36 | 1628 | -2.07 |
| 14 | [rs759168981](https://www.ncbi.nlm.nih.gov/snp/rs759168981) | C/T | P117S | 0 | 0.999 | 1 | -1.36 | 1628 | -2.39 |
| 15 | [rs773635184](https://www.ncbi.nlm.nih.gov/snp/rs773635184) | G/T | **V83F** | 0 | 0.999 | 1 | -1.03 | 1628 | -1.54 |
| 16 | [rs776743009](https://www.ncbi.nlm.nih.gov/snp/rs776743009) | G/T | G76V | 0 | 1 | 1 | -0.42 | 1628 | -1.54 |
| 17 | [rs780803760](https://www.ncbi.nlm.nih.gov/snp/rs780803760) | C/G | **F38L** | 0 | 0.999 | 0.992 | -0.19 | 1628 | -1.31 |
| 18 | [rs1028237381](https://www.ncbi.nlm.nih.gov/snp/rs1028237381) | A/C | K216Q | 0 | 1 | 0.998 | -0.46 | 1628 | -1.59 |
| 19 | [rs1160651565](https://www.ncbi.nlm.nih.gov/snp/rs1160651565) | C/G | P117R | 0 | 1 | 1 | -0.63 | 1628 | -2.42 |
| 20 | [rs1179039850](https://www.ncbi.nlm.nih.gov/snp/rs1179039850) | G/T | **V228L** | 0 | 0.997 | 0.994 | -0.45 | 1628 | -1.79 |
| 21 | [rs1203776934](https://www.ncbi.nlm.nih.gov/snp/rs1203776934) | G/A | **G103D** | 0 | 1 | 1 | -2.28 | 1628 | -2.51 |
| 22 | [rs1259762053](https://www.ncbi.nlm.nih.gov/snp/rs1259762053) | A/G | **Y295C** | 0 | 1 | 0.999 | -0.22 | 1628 | -1.96 |
| 23 | [rs1317685509](https://www.ncbi.nlm.nih.gov/snp/rs1317685509) | T>A | S240T | 0 | 0.999 | 0.995 | 0.18 | 1628 | 1.84 |
| 24 | rs1322897154 | C>T | **H307Y** | 0 | 0.998 | 0.943 | 0.15 | 1628 | -1.96 |
| 25 | [rs1392454854](https://www.ncbi.nlm.nih.gov/snp/rs1392454854) | G>C | R112T | 0 | 1 | 0.998 | -0.61 | 1628 | -2.04 |
| 26 | [rs1479729827](https://www.ncbi.nlm.nih.gov/snp/rs1479729827) | T>C | Y106H | 0 | 1 | 0.999 | -0.7 | 1628 | -2.08 |

| 27 | [rs1567384728](https://www.ncbi.nlm.nih.gov/snp/rs1567384728) | G>A | **G312S** | 0 | 1 | 0.991 | -0.43 | 1628 | -2.49 |
| --- | --- | --- | --- | --- | --- | --- | --- | --- | --- |

**Table S2:** RMSD Values of Deleterious SNPs.

| **S.No.** | **RESIDUAL**  **CHANGE** | **RMSDGROMACS**  **VALUE** | **SCHRONDIGERRMSD**  **VALUE** |
| --- | --- | --- | --- |
| 1 | P288L | 0.383 | 19.801 |
| 2 | R322Q | 67.13 | 19.6809 |
| 3 | G103D | 0.714 | 20.0964 |
| 4 | Y295C | 0.377 | 5.0827 |
| 5 | H307Y | 0.305 | 19.8465 |
| 6 | G312S | 111.9 | 19.8515 |
| 7 | V83F | 0.269 | 0 |
| 8 | F38L | 0.251 | 19.4759 |
| 9 | V228L | 0.288 | 20.8018 |
| 10 | R316Q | 0.449 | 19.3233 |
| 11 | M207V | 0.255 | 19.3084 |
| 12 | R96P | 0.544 | 18.8998 |
